# Variation in brain connectivity during motor imagery and motor execution in stroke patients based on electroencephalography

**DOI:** 10.3389/fnins.2024.1330280

**Published:** 2024-02-02

**Authors:** Dongju Guo, Jinglu Hu, Dezheng Wang, Chongfeng Wang, Shouwei Yue, Fangzhou Xu, Yang Zhang

**Affiliations:** ^1^Rehabilitation Center, Qilu Hospital of Shandong University, Jinan, Shandong, China; ^2^International School for Optoelectronic Engineering, Qilu University of Technology (Shandong Academy of Sciences), Jinan, China; ^3^Rehabilitation and Physical Therapy Department, Shandong University of Traditional Chinese Medicine Affiliated Hospital, Jinan, Shandong, China

**Keywords:** motor imagery, stroke, electroencephalography, motor execution, premotor area, primary motor cortex

## Abstract

**Objective:**

The objective of this study was to analyze the changes in connectivity between motor imagery (MI) and motor execution (ME) in the premotor area (PMA) and primary motor cortex (MA) of the brain, aiming to explore suitable forms of treatment and potential therapeutic targets.

**Methods:**

Twenty-three inpatients with stroke were selected, and 21 right-handed healthy individuals were recruited. EEG signal during hand MI and ME (synergy and isolated movements) was recorded. Correlations between functional brain areas during MI and ME were compared.

**Results:**

PMA and MA were significantly and positively correlated during hand MI in all participants. The power spectral density (PSD) values of PMA EEG signals were greater than those of MA during MI and ME in both groups. The functional connectivity correlation was higher in the stroke group than in healthy people during MI, especially during left-handed MI. During ME, functional connectivity correlation in the brain was more enhanced during synergy movements than during isolated movements. The regions with abnormal functional connectivity were in the 18th lead of the left PMA area.

**Conclusion:**

Left-handed MI may be crucial in MI therapy, and the 18th lead may serve as a target for non-invasive neuromodulation to promote further recovery of limb function in patients with stroke. This may provide support for the EEG theory of neuromodulation therapy for hemiplegic patients.

## 1 Introduction

Stroke has a high incidence and disability rate, usually with different degrees of motor dysfunction, which places a great burden on the patient’s family and society (Saini et al., 2021; Thayabaranathan et al., 2022). The recovery of motor function after stroke is characterized by a transition from a phase of soft paralysis to a period of synergy movements involving multiple muscles or muscle combinations (McPherson and Dewald, 2022), followed by a phase of isolated movements of single muscles or muscle combinations (Kuptniratsaikul et al., 2017). Brain connectivity varies at different stages of motor recovery in patients with hemiplegia (Bayrak et al., 2019).

Electroencephalography (EEG) can record functional connectivity in motor-related brain areas and predict the likelihood of motor recovery after a stroke (Hoshino et al., 2020). The PMA is located in the anterior part of the cortical motor area, anterior to the precentral gyrus. Its primary physiological function is the preparation and planning of movement, a crucial motor control component (Park et al., 2011; Wang et al., 2016). During ME, the MA receives signals from the PMA and subsequently sends commands to the muscles of the body to execute the movements, thereby controlling bodily motion (Fleischmann et al., 2014). In addition, motor cortical connectivity measured by EEG, particularly ipsilateral motor area and ipsilateral premotor area (PMA) connectivity, is strongly associated with motor deficits and improvement in post-stroke treatment; thus, it may be a useful biomarker of cortical function and plasticity (Wu et al., 2015). The reliability and spatial resolution of current source estimation are limited compared with functional magnetic resonance imaging (fMRI) or magnetoencephalography (MEG); however, EEG has the advantage of being low-cost and portable (Xiaogang et al., 2021). It can be used for bedside assessment of patients with stroke, and its millisecond temporal resolution is advantageous for exploring dynamic changes in neural activity (Warbrick, 2022).

However, the current literature on EEG studies is mostly centered on the connectivity of brain functional areas during finger tapping trials (Kim et al., 2018; Lee et al., 2021; Gyulai et al., 2023) without correlation with the stage of motor function recovery after stroke, and studies on brain motor functional areas during synergy and isolated movements are lacking.

Motor imagery (MI) therapy is a commonly used rehabilitation treatment that activates brain regions by imagining movements that help improve the functional connectivity of the brain (Monteiro et al., 2021; King et al., 2022). Currently, the majority of MI studies are conducted on the affected limb (Dai et al., 2022). However, very few studies have investigated whether left-or right-handed MI promotes greater activation of functional brain regions and stronger correlations between functional regions in patients with stroke hemiplegia. It remains inconclusive which MI program is more effective for patient recovery. Therefore, we aimed to quantify EEG signals during MI and ME and to express the correlation between different brain regions as connection strengths by calculation (Waninger et al., 2020) to explore the changes in connectivity between functional brain regions during left-and right-handed MI and different movement modes. This research could provide neural theoretical support for choosing appropriate forms of treatment and therapeutic targets for patients with stroke.

## 2 Materials and methods

### 2.1 General information

Twenty-three patients with stroke who were hospitalized in the Department of Rehabilitation of Qilu Hospital of Shandong University between March 2021 and January 2023 were selected, of whom 11 were left hemiplegic and 12 were right hemiplegic. Twenty-one healthy people, all of whom were right-handed, were also recruited. The differences between the patients with stroke and healthy controls were not significant in terms of age and sex (Table 1).

**Table 1:** General participant information.

		Left hemiplegia	Right hemiplegia	Healthy people	*P*
Sex	Male	8	8	13	0.592
	Female	3	4	8	
Age		50 ± 11.21	52.58 ± 13.54	40.71 ± 17.39	0.051
Number of cases		11	12	21	
Ischemic stroke		7	9		
Hemorrhagic stroke		4	3		
Underlying diseases (Hypertension or diabetes)		9	10	13	0.124
Heavy smoking or alcohol abuse		3	4	2	0.179
Sportsmen		2	1	5	0.594

The inclusion criteria were: (1) conformance to the “Chinese Stroke Association guidelines for clinical management of cerebrovascular disorders” (Cao et al., 2020; Liu et al., 2020) confirmed by cranial computed tomography or MRI; (2) stable vital signs, without serious cardiac, hepatic, renal, and other important organ insufficiencies; (3) the first occurrence of cerebral hemorrhage, cerebral infarction, Brunnstrom staging >4, disease duration <1 year; (4) clear mental state, with imaginative ability, no cognitive dysfunction, and a simple Mental State Examination score > 25 points; and (5) signed the informed consent for treatment by patients or family members.

The exclusion criteria were: (1) unstable condition, in acute progressive stage or complication of serious heart, liver, kidney diseases, and infections; (2) severe cognitive dysfunction, mental disorder, or uncooperative; (3) presence of unilateral neglect and apraxia; (4) illiteracy, low vision, or blindness; and (5) metal implants in the head. This study was approved by the Ethical Review Committee of Qilu Hospital of Shandong University [Approval No. KLY-2020 (KS)-477].

### 2.2 Methods

#### 2.2.1 EEG signal acquisition

**Figure 1:**
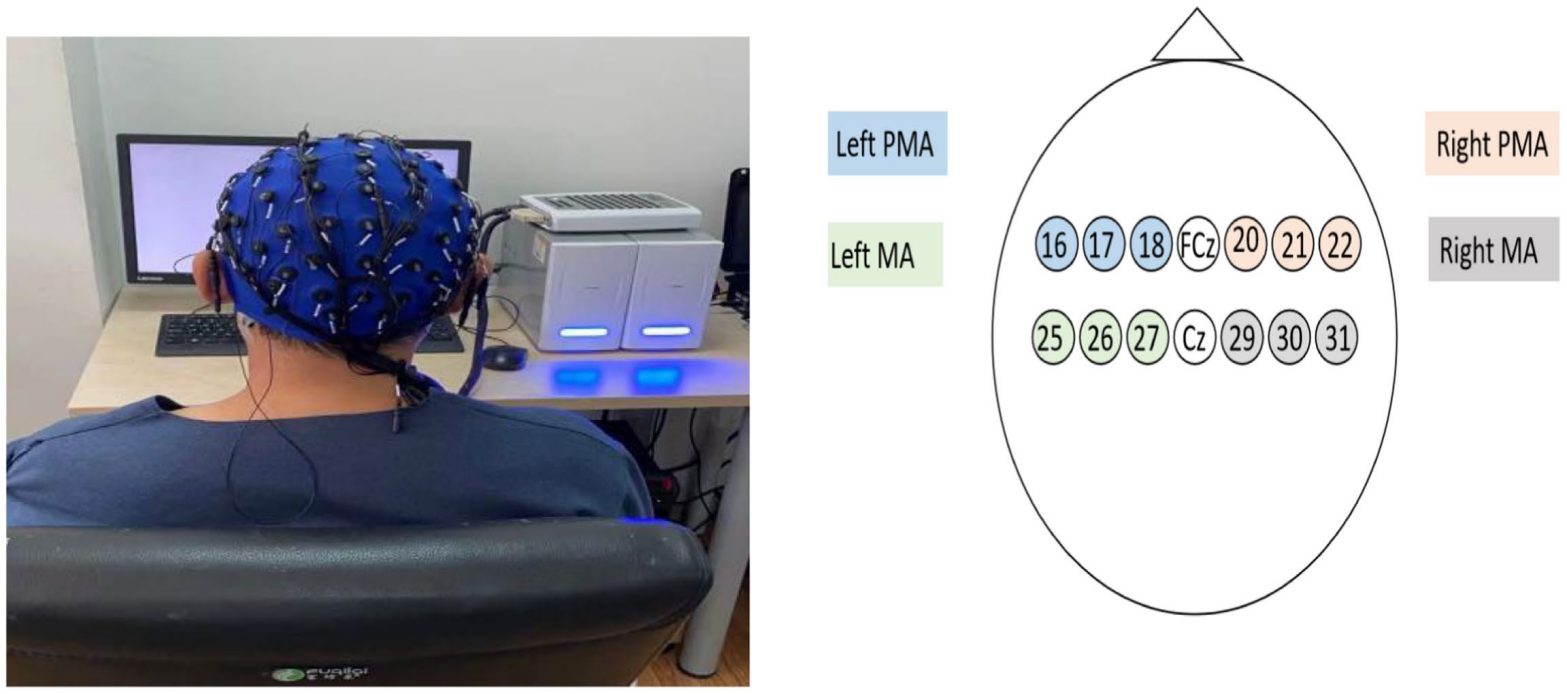
EEG acquisition process and lead composition of bilateral PMA and MA areas.

EEG acquisition and positioning were performed according to the international 10–20 standard, using 64-lead EEG electrodes for signal acquisition. Two regions of interest were studied: the PMA and the primary motor cortex (MA). Activation of the PMA (L-PMA/R-PMA) consisted of leads 16 (FC5), 17 (FC3), 18 (FC1), 20 (FC2), 21 (FC4), and 22 (FC6), respectively, whereas activation of the MA region (L-MA/R-MA) consisted of leads 25 (C5), 26 (C3), and 27 (C1) and 29 (C2), 30 (C4), and 31 (C6), respectively (Figure 1).

#### 2.2.2 Recording paradigm

This experiment consisted of three parts. The first part was MI, including three modules. Each module included 20 randomized left-or right-handed MIs. Each trial was recorded for 6 s with a 4-s interval, and a 90-s rest was taken after the completion of each module, for a total of 60 trials, including 30 left-handed MIs and 30 right-handed MIs. In the second part, the hemiplegic hand was used to perform the movement: “touching your lower lip with the thenar of your hand” (movement 1 [M1]), with a recording time of 15 s for each movement, 15 s apart, and a total of five repetitions. The third part involved using the hemiplegic hand to perform “grasping dowels” (movement 2 [M2]). Each movement was repeated five times, with the same recording and interval time as that of M1. Healthy individuals performed both movements using their right hand. The entire experiment lasted for approximately 20 min. All electrodes were appropriately filled with conductive gel throughout the experiment to ensure all impedances remained below 10 KΩ. Movements 1 and 2 represent typical synergy and isolated motion patterns, respectively (Pan et al., 2018).

#### 2.2.3 EEG data processing

The selection of α (8–12 Hz) and β (13–30 Hz) bands has been reported in previous studies (Choy et al., 2023 Lakshminarayanan et al., 2023). The data were pre-processed by filtering (8–30 Hz) and down-sampling to 100 Hz, and an independent component analysis was performed to improve the EEG signal quality. Using the modified S-Transform (MST), the Power Spectral Density (PSD; the power of the EEG signal in one unit frequency band, indicating that the signal power varies with frequency) was calculated, and the PSD on each channel was normalized and summed. The formula was as follows:


$$MST\left(\tau ,\;f\right)=\int_{-\infty }^{\infty }x\left(t\right)g\left(\tau -t,\;f\right)e^{\left(-j2\pi ft\right)}dt$$

$$x_{n}=\frac{x-\min}{\max-\min}$$

In addition, we assessed the strength of the functional connectivity between channels by comparing the magnitudes of the correlation coefficients between different channels (Lin et al., 2020; Zekun et al., 2021; Shanshan et al., 2023) and presented them in the form of heatmaps.

#### 2.2.4 Statistical analysis

Analyses were performed using SPSS version 25 (IBM Corp., Armonk, NY, USA), and *p* ≤ 0.05 (two-tailed) was considered statistically significant. The measurement information was expressed as mean ± standard deviation. The various types of measurement data satisfied the normal distribution and chi-square, and the differences between and within groups were compared using the paired sample *t*-test and independent sample *t*-test, respectively. Chi-square test was used for categorical variables. Using GraphPad Prism 8 Ink software (GraphPad, San Diego, CA, USA), Pearson’s correlation analysis was used to compare the correlation between the EEG signal characteristics of the PMA and the corresponding MA areas.

## 3 Results

### 3.1 PMA and MA are significantly positively correlated with the MI hand

**Figure 2:**
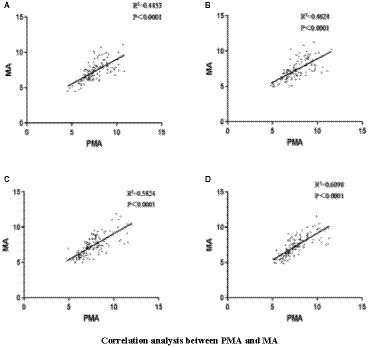
**(A)** Left hand MI in healthy individuals; **(B)** Right hand MI in healthy individuals; **(C)** the affected hand MI in patients with stroke; **(D)** the unaffected hand MI in patients with stroke.

Pearson’s correlation analysis revealed that when healthy individuals performed left-or right-handed MI, the MA EEG signals increased with the increase in PMA EEG signals, and the two were linearly correlated (Figures 2A,B). When patients with stroke performed MI of the affected or unaffected hand, the results were the same as in healthy individuals (Figures 2C,D), suggesting that hand MI promotes the increase of MA EEG signals in patients with stroke and healthy individuals.

### 3.2 The correlation coefficient between PMA and MA was higher in left-hand MI than in right-hand MI in both healthy people and patients with stroke

**Figure 3:**
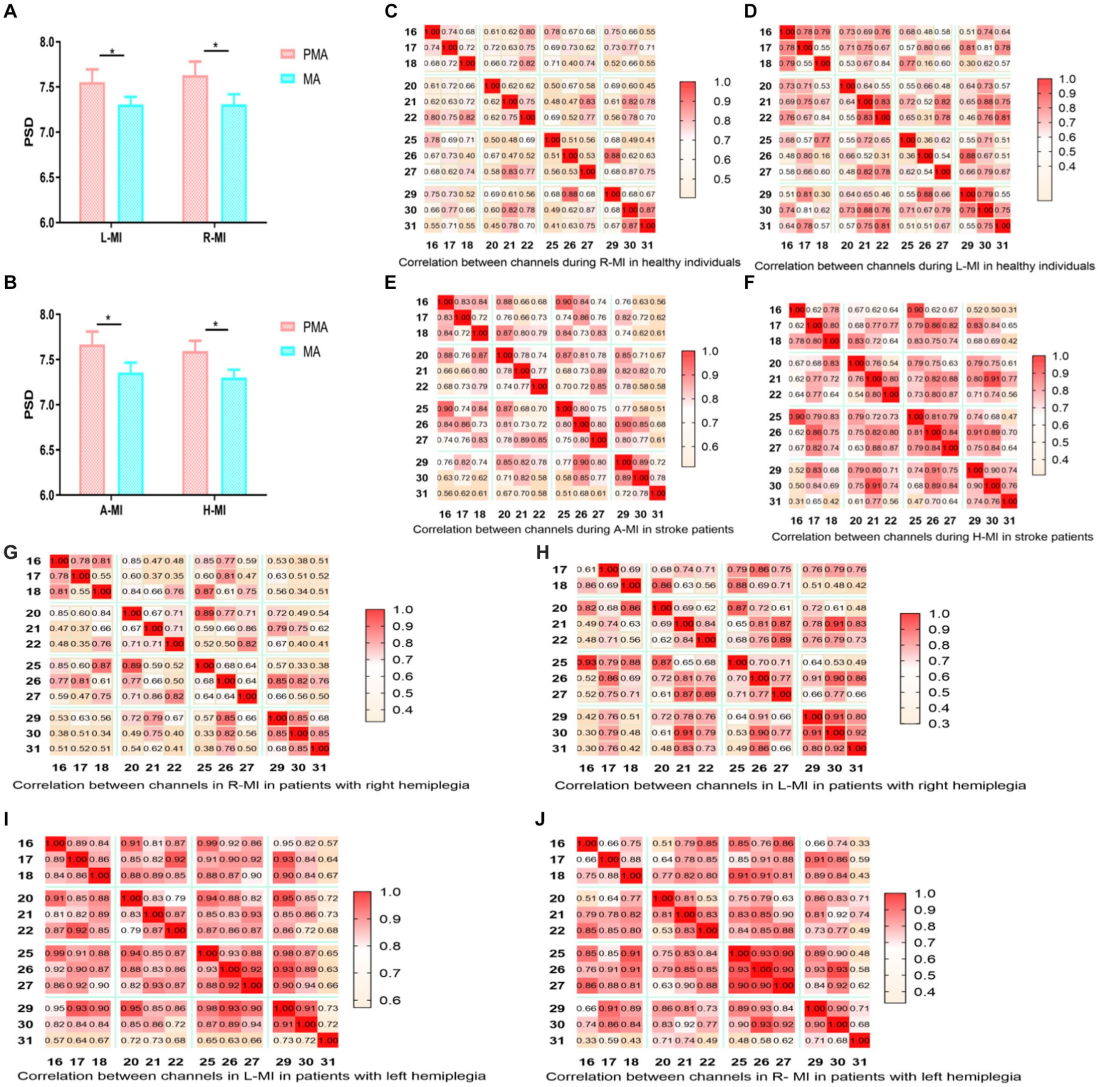

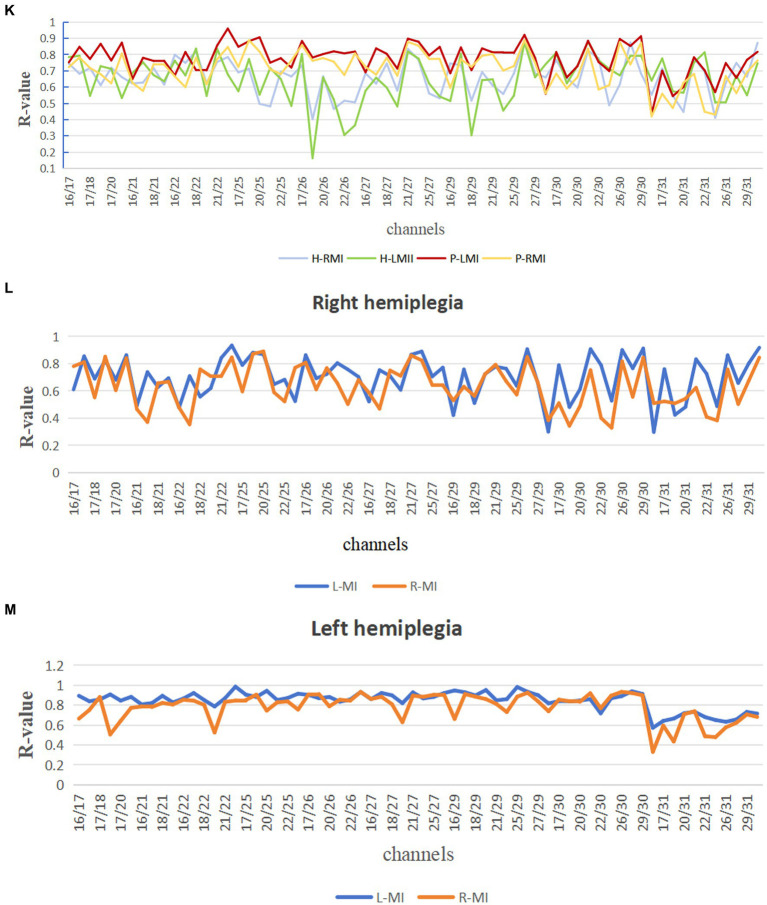
Comparison of PMA and MA in hand MI and functional connection diagram between channels. Comparison between PMA and MA during left-and right-handed MI in healthy participants; **(B)** Comparison between PMA and MA during MI in the affected and unaffected hands in patients with stroke; **(C–J)** The correlation between 12 channels during right-handed MI, left-handed MI, affected-hand MI in patients with stroke, unaffected-hand MI in patients with stroke, right-handed MI in right patients with hemiplegia, left-handed MI in patients with right hemiplegia, left-handed MI in patients with left hemiplegia, and right-handed MI in patients with left hemiplegia. **(C–J)** Horizontal and vertical coordinates indicate the number of channels; squares indicate correlation coefficients between channels; color change represents the strength of the correlation coefficients. PMA, premotor area; MA, motor area; L-MI, left-hand motor imagery; R-MI, right-hand motor imagery; A-MI, motor imagery of the affected hand; H-MI, motor imagery of the unaffected hand. **(K–M)** Comparison of the magnitude of correlation coefficients between channels during left-and right-handed MI in stroke patients and healthy individuals; right hemiplegic patients and left hemiplegic patients. H-RMI, right-handed MI for healthy individuals; H-LM, left-handed MI for healthy individuals; P-LMI, left-handed MI for stroke patients; P-RMI, right-handed MI for stroke patients. *p < 0.05.

The PSD values of the EEG signals in the PMA were significantly greater than those in the MA in both healthy individuals and patients with stroke, regardless of the side (left or right) (*p* < 0.01) (Figures 3A,B). When healthy people and patients with stroke performed hand MI, each node was correlated with the other 11 nodes, and the color change represents the strength of the correlation coefficient, with light yellow being the weakest and dark red being the strongest. The brain functional connectivity maps were plotted according to the magnitude of Pearson’s correlation coefficient R-value (Figures 3C–F; Cao et al., 2022). In healthy individuals, the strength of brain functional connectivity was higher in left-handed MI than in right-handed MI, and the results were the same in stroke patients regardless of right-sided or left-sided hemiparesis (Figures 3G–JL–M). The functional connectivity correlation was higher in the stroke group than in healthy people during MI, especially during left-handed MI (Figure 3K).

### 3.3 Correlation coefficients between PMA and MA were higher in synergy movements than in isolated movements in patients with stroke

**Figure 4:**
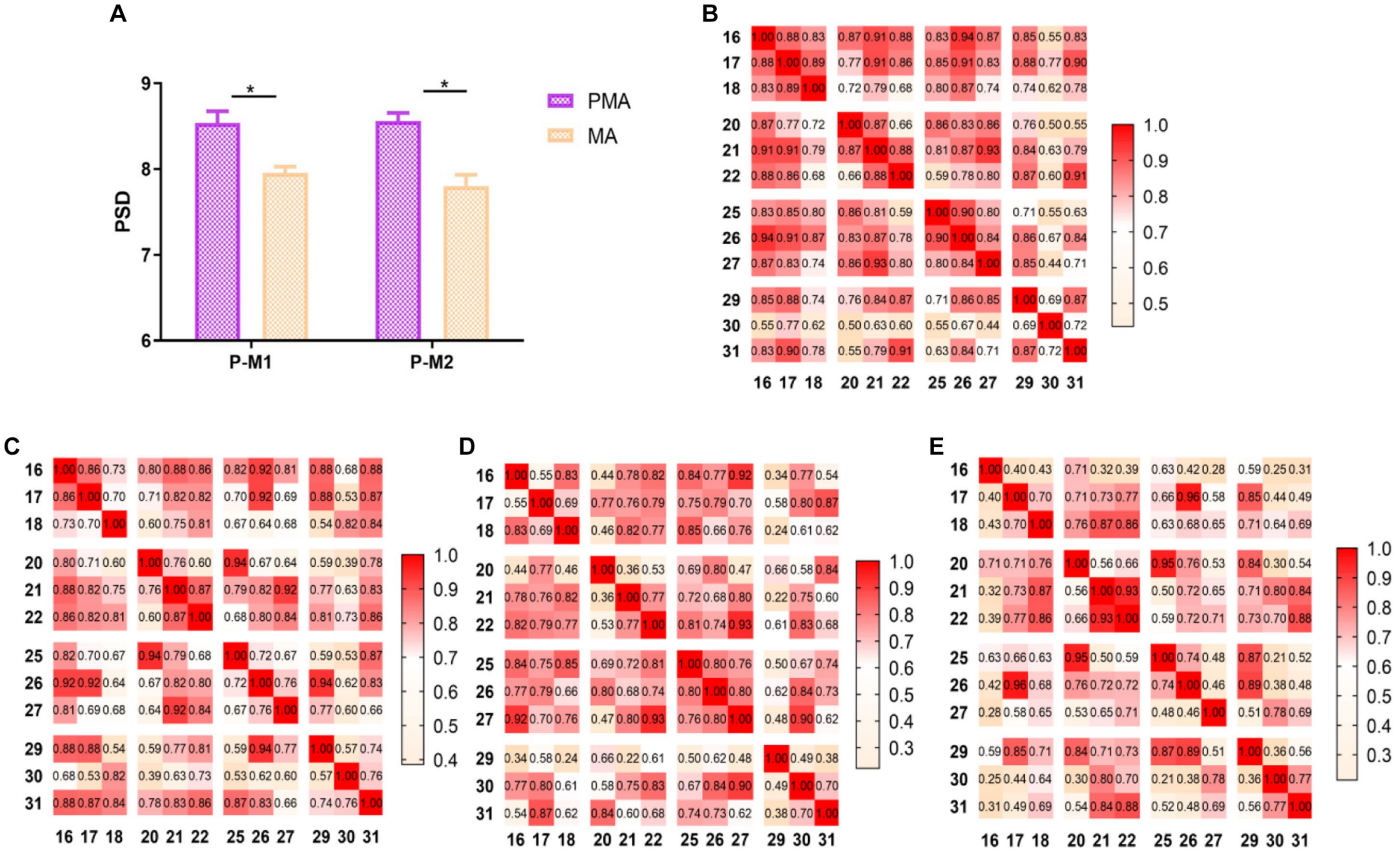
Comparison of PMA and MA in ME and functional connection diagram between channels. **(A)** Comparison between PMA and MA in patients with stroke during synergy and isolated movements, respectively; **(B)** correlation between 12 channels in synergy movements in patients with right hemiplegia; **(C)** correlation between 12 channels in isolated movements in patients with right hemiplegia; **(D)** correlation between 12 channels in synergy movements in patients with left hemiplegia; **(E)** correlation between 12 channels in isolated movements in patients with left hemiplegia; **(B–E)** horizontal and vertical coordinates indicate the number of channels; squares indicate the correlation coefficients between channels; color changes represent the strength of the correlation coefficients. *p < 0.05.

The PMA PSD values were also significantly greater than the MA PSD values when patients with stroke performed both synergy and isolated movements (*p* < 0.01) (Figure 4A). Brain functional connectivity maps were plotted when patients with stroke performed synergy and isolated movements (Figures 4B–E), in which the light-yellow color correlation was the weakest and the dark-green color was the strongest. The results revealed that the connection strength between the PMA and MA of the brain was higher during synergy movements than during isolated movements, regardless of whether the patient was right or left hemiplegic.

### 3.4 Functional connectivity strength was highest in channel 18 (FC1) during action execution between patients with right hemiplegia and healthy individuals

**Figure 5:**
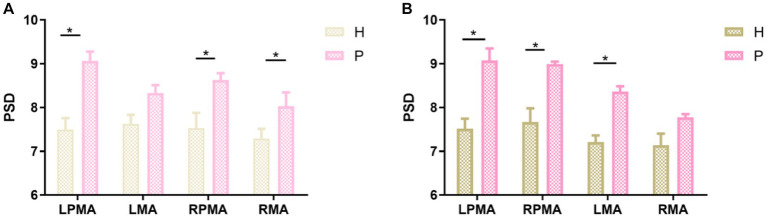
Comparison between the same brain regions during action execution. **(A)** Comparison between the brain regions of healthy individuals and patients with stroke during synergy movements; **(B)** comparison between the brain regions of healthy individuals and patients with stroke during isolated movements. H, healthy individuals; P, patients with stroke. *p < 0.05.

Regardless of whether the action was a synergy movement (Figure 5A) or isolated movement (Figure 5B), the PSD values of patients with right hemiplegia were higher than those of healthy individuals in LPMA, RPMA, LMA, and RMA, especially in LPMA and RPMA, and the differences were significant between the groups (Figure 5A; LPMA, *p* < 0.01; RPMA, *p* < 0.05) (Figure 5B; LPMA, RPMA, *p* < 0.01), suggesting that the sensitivity of PMA was higher than that of MA during ME in patients with stroke. Therefore, we only compared the PSD values between each channel of the PMA and the other channels. During synergy movement, nine channels were significantly different from channel 18, and four channels were significantly different from channels 17, 20, and 22, respectively (Table 2). During isolated movements, ten channels were significantly different from channel 18, and eight channels were significantly different from channels 21 and 22, respectively (Table 3). Therefore, channel 18 had the largest number of differences from the other channels, suggesting that the main area of PMA functional connectivity differences during synergy and isolated movements in patients with stroke was the area distributed by channel 18. During isolated movements, channels 21 and 22 also exhibited more significant differences.

**Table 2:** Differences in PSD between the channels in the two groups based on synergy movements.

Channel	*P*	*H*	*T*	*p*
17–21	9.28 ± 3.13	6.96 ± 1.37	2.435	0.029
17–27	9.28 ± 3.13	7.22 ± 1.38	2.164	0.049
17–29	9.28 ± 3.13	6.97 ± 1.32	2.435	0.030
17–30	9.28 ± 3.13	7.20 ± 1.55	2.150	0.049
18–16	9.28 ± 2.23	7.36 ± 1.49	2.970	0.006
18–17	9.28 ± 2.23	7.12 ± 1.87	2.968	0.006
18–21	9.28 ± 2.23	6.96 ± 1.37	3.713	0.001
18–22	9.28 ± 2.23	7.47 ± 1.66	2.666	0.012
18–25	9.28 ± 2.23	7.81 ± 1.63	2.173	0.038
18–27	9.28 ± 2.23	7.22 ± 1.38	3.294	0.002
18–29	9.28 ± 2.23	6.97 ± 1.32	3.758	0.001
18–30	9.28 ± 2.23	7.20 ± 1.55	3.149	0.004
18–31	9.28 ± 2.23	7.71 ± 1.48	2.436	0021
20–17	8.76 ± 2.63	7.12 ± 1.87	2.083	0.046
20–21	8.76 ± 2.63	6.96 ± 1.37	2.207	0.044
20–29	8.76 ± 2.63	6.97 ± 1.32	2.207	0.044
20–30	8.76 ± 2.63	7.20 ± 1.55	2.149	0.040
22–17	8.81 ± 2.77	7.12 ± 1.87	2.086	0.045
22–21	8.81 ± 2.77	6.96 ± 1.37	2.166	0.048
22–29	8.81 ± 2.77	6.97 ± 1.32	2.166	0.048
22–30	8.81 ± 2.77	7.20 ± 1.55	2.145	0.040

**Table 3:** Differences in PSD between channels in the two groups based on isolated movements.

Channel	*P*	*H*	*T*	*p*
16–22	8.80 ± 2.46	7.27 ± 1.42	2.282	0.030
16–26	8.80 ± 2.46	7.22 ± 1.92	2.053	0.049
16–27	8.80 ± 2.46	6.94 ± 1.63	2.167	0.014
16–29	8.80 ± 2.46	6.70 ± 1.43	3.124	0.004
16–30	8.80 ± 2.46	7.10 ± 1.44	2.514	0.017
17–26	8.78 ± 3.03	6.94 ± 1.63	2.286	0.029
17–27	8.78 ± 3.03	6.70 ± 1.43	2.696	0.011
17–29	8.78 ± 3.03	7.10 ± 1.44	2.167	0.038
18–16	9.63 ± 2.76	7.25 ± 2.00	2.865	0.007
18–20	9.63 ± 2.76	7.45 ± 1.67	2.849	0.008
18–21	9.63 ± 2.76	7.27 ± 1.42	2.762	0.015
18–22	9.63 ± 2.76	7.47 ± 2.14	2.515	0.017
18–25	9.63 ± 2.76	7.22 ± 1.92	2.952	0.006
18–26	9.63 ± 2.76	6.94 ± 1.63	3.535	0.001
18–27	9.63 ± 2.76	6.70 ± 1.43	3.426	0.004
18–29	9.63 ± 2.76	7.10 ± 1.44	2.950	0.010
18–30	9.63 ± 2.76	7.61 ± 1.42	2.363	0.033
18–31	9.63 ± 2.76	7.32 ± 1.61	2.657	0.018
20–27	8.91 ± 3.05	6.70 ± 1.43	2.364	0.033
21–16	9.00 ± 2.17	7.25 ± 2.00	2.341	0.026
21–20	9.00 ± 2.17	7.45 ± 1.67	2.300	0.028
21–25	9.00 ± 2.17	7.22 ± 1.92	2.433	0.021
21–26	9.00 ± 2.17	6.94 ± 1.63	3.082	0.004
21–27	9.00 ± 2.17	6.70 ± 1.43	3.670	0.001
21–29	9.00 ± 2.17	7.10 ± 1.44	3.011	0.005
21–30	9.00 ± 2.17	7.61 ± 1.42	2.217	0.034
21–31	9.00 ± 2.17	7.32 ± 1.61	2.534	0.017
22–16	9.09 ± 2.71	7.32 ± 1.61	2.363	0.025
22–17	9.09 ± 2.71	7.25 ± 2.00	2.231	0.033
22–21	9.09 ± 2.71	7.45 ± 1.67	2.162	0.038
22–26	9.09 ± 2.71	7.22 ± 1.92	2.307	0.028
22–27	9.09 ± 2.71	6.94 ± 1.63	2.851	0.008
22–29	9.09 ± 2.71	6.70 ± 1.43	3.330	0.002
22–30	9.09 ± 2.71	7.10 ± 1.44	2.757	0.010
22–31	9.09 ± 2.71	7.61 ± 1.42	2.063	0.048

## 4 Discussion

In this study, we investigated the characteristics and differences in brain functional connectivity in the PMA and MA during MI and ME in patients with stroke and healthy individuals using EEG. The results revealed that PMA activation was more evident during MI and ME in both groups. The functional connectivity correlation was higher in the stroke group than in healthy participants during MI and was obvious during left-handed MI in both groups. The functional connectivity correlation was enhanced in synergy movement compared with isolated movement during ME, and all regions with abnormal functional connectivity were in the 18th lead of the left PMA region.

### 4.1 PMA plays a significant role in MI and ME

MI is a repetitive motion imagination based on motor memory that activates a specific brain area for an activity (Chepurova et al., 2022). The PMA of the brain sends signals to the MA to simulate movement. This imagery process can help people better prepare for the actual movement. Therefore, PMA is more obviously activated than MA during MI (Matsuo et al., 2021; Ma et al., 2022; Zou et al., 2022). The activity of the MA may be driven by the facilitating or inhibiting effect of the PMA. With the enhancement of the EEG signals of the PMA, the EEG signals of the MA increase, and the two areas exhibit a significant positive correlation. This is consistent with our findings. Some studies have revealed that the relationship between the strength of the PMA-MA connections within the affected cortex and motor performance is not invariable. In the subacute phase, the connection strength is negatively correlated with motor function, whereas in the chronic phase, these factors are positively correlated (Lam et al., 2018; Paul et al., 2021). Zhe et al. (2020) chose high-frequency repetitive transcranial magnetic stimulation (TMS) to act on the PMA of patients and improve the recovery of motor function in the upper limbs and hands. A study combining fMRI revealed that cortical plasticity in the MA area was relatively spatially limited after TMS treatment, whereas cortical remodeling occurred in the PMA (Tamura et al., 2019) suggesting that the PMA is partially involved in functional recovery. In addition, the PMA continuously receives feedback from the MA, such as muscle tension and the effects of previously executed movements, which further influence and adjust the execution of movements. Our study revealed that PMA activation was greater in patients with stroke during the execution of synergy or isolated movements. This implies that in patients with stroke hemiparesis, when the brain’s normal motor control and coordination are disrupted due to damage to the brain’s motor pathways, the PMA, responsible for motor planning, preparation, and control, needs to be more strongly activated to facilitate the MA in producing different movements. Previous studies have also pointed out that the primary function of the premotor cortex is to lay the groundwork for the motor cortex by enhancing and expediting certain movements (Fine and Hayden, 2022). Therefore, we can infer that the PMA plays a more crucial role in motor execution in hemiplegic patients. In addition, some studies have reported that activation during dominant hand movements is relatively greater than that during non-dominant hand movements (Wu et al., 2017). Therefore, in this study, we also compared the EEG signals between brain regions during the execution of right-handed movements in healthy participants and patients with right hemiplegia and discovered that the activation of the movement-related brain regions was greater than that of healthy participants during the execution of movements in patients with hemiplegia and that the activation of the PMA was more evident, similar to the findings of the study conducted by Chunyong et al. (2023). PMA plays an important role in controlling synergy and isolated movements. Finally, we compared the PSD values of EEG signals between each channel in the PMA area and other channels. We discovered that channel 18 had the largest number of differences from the other channels, suggesting that the main area of PMA functional connectivity differences during synergy and isolated movements in patients with stroke was the area distributed by channel 18.

### 4.2 Correlations between functional brain regions are stronger in left-handed MI

The brain is a complex network system. Activities in the PMA not only drive activities in the MA, but activities in the MA also drive activities in the PMA, and they interact with each other (D'Aleo et al., 2022). Therefore, we have conducted an analysis of the functional connectivity of different channels in the brain’s PMA and MA. Previous research (Bajaj et al., 2015; Park et al., 2015; Mencel et al., 2022; Riahi et al., 2023) has demonstrated that MI therapy can improve motor skills, promote brain plasticity, and repair damaged neural connections. However, the extent to which connectivity within the brain hemispheres is associated with left-and right-handed MI, as reported in the literature, is inconsistent. Liu et al. (2017) demonstrated that differences exist in the activation of functional brain areas between left-and right-handed MI. Leeuwis et al. (2021) found that the right hemisphere’s alpha band connectivity was significantly improved in MI tasks.

Our previous study revealed that MI activates both the PMA and MA areas and that PMA activation is significantly higher than MA activation during left-handed MI (Dongju et al., 2023). In this study, the connectivity between the functional areas of the brain was stronger in left-handed MI than in right-handed MI, which may be related to the different divisions of labor between the left and right hemispheres. The left hemisphere is responsible for logic, comprehension, memory, analysis, and language, whereas the right hemisphere is mainly responsible for spatial imagery and plays a major role in the imagination of art or music. Thus, left-handed MI may be more effective at promoting limb function recovery.

### 4.3 Connections between functional areas of the brain are more obvious in synergy movement than in isolated movement in patients with stroke

We compared the two phases of synergy and isolated movement during the process of motor recovery in patients with stroke with hemiplegia. In synergy movements, both the motor cortex and various movement-related areas of the brain are activated to orchestrate the control of multiple joint and muscle groups. As the transition occurs from synergy to isolated movements, specific joints and muscles can be engaged for partial movement. In isolated movements, only the region in the motor cortex responsible for that particular action needs to be activated to execute the movement. Therefore, the cerebral cortex regions activated during these two exercise modes differ (Cheng et al., 2021). Synergy movement involves more areas of the cortex and requires more complex neural control and precise coordination (Maier et al., 2019). Our results suggest that the connections between functional brain regions are stronger during synergy than during isolated movements. Hence, during non-invasive neuromodulation, the choice of stimulation range should align with the hemiplegic movement pattern. When a patient is in the synergy movement phase, a larger stimulation range is appropriate, whereas during the isolated movement period, a smaller stimulation range is advisable.

### 4.4 Limitations

Our study had some limitations. Due to the small sample size, the test site was limited to the PMA and MA of the brain. Therefore, in the future, we should expand the sample size, increase the test range, and conduct an in-depth study to provide a neuroelectrophysiological basis for characterizing the recovery of motor function in patients with stroke.

## 5 Conclusion

In conclusion, left-handed MI is preferred when providing MI therapy to patients with stroke hemiplegia. Motor function recovery may be enhanced when targeting the 18^th^ lead of the left PMA area for stimulation. Our results provide theoretical support for the choice of parameters for stroke rehabilitation.

## Data availability statement

The raw data supporting the conclusions of this article will be made available by the authors, without undue reservation.

## Ethics statement

The studies involving humans were approved by Shandong University Qilu Hospital Research Ethics Committee. The studies were conducted in accordance with the local legislation and institutional requirements. The participants provided their written informed consent to participate in this study. Written informed consent was obtained from the individual(s) for the publication of any potentially identifiable images or data included in this article.

## Author contributions

DG: Writing – original draft, Writing – review & editing. JH: Writing – original draft. DW: Formal analysis, Writing – review & editing. CW: Data curation, Formal analysis, Writing – review & editing. SY: Writing – review & editing. FX: Writing – review & editing. YZ: Writing – review & editing.
